# The expanded program on immunization service delivery in the Dschang health district, west region of Cameroon: a cross sectional survey

**DOI:** 10.1186/s12889-016-3429-7

**Published:** 2016-08-17

**Authors:** Walter Ebile Akoh, Jérôme Ateudjieu, Julienne Stephanie Nouetchognou, Martin Ndinakie Yakum, Fabrice Djouma Nembot, Sonia Nafack Sonkeng, Micheal Saah Fopa, Pierre Watcho

**Affiliations:** 1 Department of Biomedical Sciences, University of Dschang, PO Box 067, Dschang, Cameroon; 2 Division of Health Operations Research, Ministry of Public Health, Yaoundé, Cameroon; 3 Meilleure Accés aux Soins de Santé (M.A SANTE), PO Box 33490, Yaoundé, Cameroon

**Keywords:** EPI, Immunization, Service delivery, Dschang health district, Cameroon

## Abstract

**Background:**

Vaccination is the most effective intervention strategy, and the provision of vaccination at fixed posts and outreach posts is a backbone of a sustainable vaccination system in developing countries. Access to immunization services is still limited in Cameroon. Several health districts in the west region have recorded new epidemic outbreaks, including the occurrence of a wild polio virus epidemic outbreak in 2013. The aim of this study was to assess immunization service delivery in one of the largest health districts in the west region of Cameroon; the Dschang Health district.

**Methods:**

It was a cross sectional study conducted in 2013, in 42 health facilities covering 18 health areas in the Dschang Health District. Data were collected with questionnaires administered to health personnel face to face and an observation grid was used to assess resources and tools. Data were entered and analyzed in Epi Info.

**Results:**

A total of 42 health facilities were assessed and 77 health personnel were interviewed. Overall, 29 (69.0 %) health facilities organized one vaccination session monthly, 2 (4.8 %) organized an outreach within the last 3 months prior to the study, 15 (35.7 %) did not have a vaccination micro plan, 24 (32.9 %) health personnel had not been supervised for at least the last 6 months prior to the study, 7 (16.7 %) health facilities did not have a functional refrigerator, 1 (2.4 %) did not have a vaccine carrier, 23 (54.8 %) did not have a means of transport (vehicle or motorcycle) and 12 (28.6 %) did not have an EPI guideline. The knowledge of health personnel on vaccine and cold chain management, and on diseases of the EPI under epidemiological surveillance was found to be limited.

**Conclusion:**

The frequency and strategic provision of immunization services in the Dschang Health district is inadequate. Resource availability for an adequate provision of immunization services is insufficient. The knowledge of health personnel on vaccine management, cold chain management and on diseases under surveillance by the EPI is limited.

**Electronic supplementary material:**

The online version of this article (doi:10.1186/s12889-016-3429-7) contains supplementary material, which is available to authorized users.

## Background

Immunization is one of the most effective intervention strategies to reduce maternal and child morbidity and mortality [1, 2]. In recognition of the benefits of immunization, there has been need to extend its coverage worldwide, especially in low income countries with the highest child and maternal mortality rates. Since its launching in 1974 [3], vaccination has contributed tremendously in reducing morbidity and mortality worldwide [4].

The Expanded Program on Immunization (EPI) was introduced in Cameroon in 1976 [5]. Since 2010, the national targets have been to achieve a national coverage of 90, and 80 % at the level of health districts [6]. To achieve these objectives, several vaccination strategies have been adopted, and recommended to be used in health facilities. These strategies includes; organizing vaccination at fixed posts, outreach vaccinations, mobile and supplementary vaccination activities [7].

Despite adoption of these strategies, in an attempt to achieve a universal coverage in the EPI, access of populations to vaccination services remains low. In fact, based on the WHO definition, only 53 % of children aged between 12 and 23 months were completely vaccinated, 5 % did not receive any antigen of the EPI, 42 % were only partially vaccinated and 5 out of the 9 antigens had national coverage rates lower than 80 %, far below the national targets [8].

Several health districts in the west region of Cameroon have recorded new cases of measles epidemic in the last few years, including the isolation of a wild poliovirus (WPV) type 1 from two acute flaccid paralysis (AFP) cases in October 2013 [9], representing the first polio-outbreak reported in the country since 2009. While it is important for the populations to collaborate with health providers by accepting vaccines, the quality of vaccination services provided by health personnel is indispensable for the success of the vaccination program. Ensuring quality provision of vaccination requires among others to; maintaining a functional cold chain to ensure vaccine potency, adequate vaccination practices, good knowledge of health personnel on targeted diseases, continuous surveillance of targeted diseases and the regular and strategic programing of vaccination sessions. Few studies have assessed the quality of the cold chain and other aspects of the immunization program, in the west region and in other parts of Cameroon [10–14], but very little is known about adherence of personnel to national guidelines on good immunization service delivery practices, and also on the knowledge of health personnel on diseases targeted by the EPI program. To fill this gap, we conducted a hospital-based cross-sectional survey in the Dschang health district (West Region, Cameroon). The objective of this study was to assess immunization service delivery, by assessing health personnel adherence to EPI guidelines regarding the vaccination strategies adopted. The study also assessed the knowledge of health personnel on diseases targeted by the EPI and the availability of resources and tools necessary for an adequate and strategic provision of vaccination services. The findings of this study will serve to guide future national vaccination policies, and also serve as a base for future studies in order to improve immunization coverage and eventually to prevent new epidemic outbreaks.

## Methods

### Study design

It was a cross sectional study, conducted in 42 health facilities from 18 selected health areas in the Dschang health district in 2013. The frequency and strategy of organizing vaccination sessions, the availability of resources and tools required for vaccination activities and the knowledge of health personnel on disease of the EPI were assessed in each health facility. Data were collected with the aid of questionnaires administered to health personnel by interview and an observation grid was used to assess tools.

### Study area

This study was conducted in the Dschang Health district, located in the west region of Cameroon. Health care delivery system in Cameroon has as objective to render Primary Health Care (PHC) accessible to the entire population through decentralization of the health management process to the health district level [15]. The health system is organized in three levels; the central, the regional and the Health District. Health policies and strategies are elaborated at the central level and implemented at the district level by the district health Service (DHS). Resources are mainly allocated by the state budget, local communities, international and national organizations.

The health district is a geographic area that covers a population between 30,000 to 400,000 inhabitants. It is divided in to health areas covering 5000 to 30,000 inhabitants. In each health area, the integrated Health Centre (IHC) is in charge of providing the Minimum Package of Activities (MPA) (smallest package of health care activities provided by health centers at the lowest level of the health system). The implementation of EPI is part of this package. This includes storing vaccines as recommended by the EPI guideline, organizing and reporting vaccination sessions, and conducting epidemiological surveillance of disease targeted by EPI. Immunization services are offered through fixed vaccination posts in health facilities, through outreach vaccinations, and through mobile vaccination strategies, based on the proximity of the target populations to the health facility. While every health facility is considered a fixed post, outreach and mobile strategies are used to reach populations father than 5 and 10 km respectively from the closest health facility. Supplementary vaccination sessions are also organized depending on emergencies such as during epidemic outbreaks. The number of personnel in each vaccination team depends on the attendance or turn out rate. For high attendance, it is comprised of a team of three personnel (the vaccinator, social mobilizer and the screener or controller) while for low attendance, a vaccination team is comprised of a team of two (the vaccinator and social mobilizer).

The Dschang health district is one of the largest districts in the west region of Cameroon, covering a surface area of about 1.060Km^2^. It is comprised of 22 health areas, and 54 health facilities, with a 2012 total population of 218 006 people; 8284 children between 0 and 11 months, and 36,843 children between 0 and 59 months. Regarding the availability of human resources, it has a total of 182 staff including 124 health personnel. More than 70 % of the EPI target populations live within 5 km to a health facility, about 20 % live within 5 to 20 Km and about 2 % live farther than 20Km from a health facility. In 2013, the Dschang health district recorded vaccination coverage of 84 % while 73 % of children had received all antigens before the age of 1 year as recommended by the guideline [14]. It is a semi urban district, with a typical relief feature characterized with hills and slopes, with a poor road infrastructure that renders geographical accessibility difficult. This situation is worse during raining seasons characterized with heavy downpours that make the roads muddy, slippery and almost inaccessible. The characteristic of the Dschang health district makes it suitable for this study.

### Sampling

Eighteen health areas were systematically selected from an exhaustive list of 22 health areas that form the Dschang Health district. In each selected health area, all health facilities, of all categories and types, that offer immunization services were eligible to participate in the study. In each health facility, one or two health personnel who are normally involved in immunization service delivery activities were interviewed. Health facilities that did not offer immunization services were excluded, and health personnel that did not consent to participate in the study were also excluded and replaced by another health personnel in the same health facility.

### Data collection

Data were collected in May 2013 by ten teams of surveyors headed by a field supervisor. In each health facility, authorization from the head of the facility was obtained, personnel involved in immunization service delivery activities were identified, consented and a questionnaire was administered face to face. Health personnel were selected at random for interview. For health facilities with only one personnel involved with immunization activities, the personnel was automatically selected and interviewed. The aim of the questionnaire was to assess the knowledge of the health personnel on diseases targeted by the expanded program on immunization, diseases under epidemiological surveillance, cold chain and vaccine management, and the frequency and strategic planning of vaccination sessions. The availability of essential resources and tools that are indispensable to ensure an adequate and strategic immunization service delivery were assessed with an observation grid and a questionnaire. During observation, the following were verified; the availability of a functional refrigerator, presence of functional thermometer in the refrigerator, availability of ice packs, existence of a temperature chart, availability of a vaccination micro plan, availability of a contingency plan, availability of a vaccine management register, presence of electricity, and availability of EPI guideline. Observation involved the visual verification of the parameter being investigated by the surveyor and the recording of what has been observed on a grid essentially made of check boxes.

#### Data management

Initially, data collection tools were pretested prior to the study. Completed questionnaires were verified for completeness and correctness by the field supervisor. Data was double entered in Epi Info and compared for differences. Logical errors were corrected. Analysis was done in Epi Info. Data analysis was essentially by calculating proportions and 95 % confidence interval. Chi square test was used to compare proportions and the importance of the difference was measured by calculating *p*-value.

## Results

### Characteristics of health facilities and health personnel

A total of 18 out of the 22 health areas were surveyed, in which 43 health facilities were eligible and 42 (97.7 %) participated. Among these, 30 (69.8 %) were public, 11 (25.5 %) private and 2 (4.7 %) were confessional health facilities. These included a district hospital, 37 (88.1 %) integrated health centers and 4 (9.5 %) sub-divisional hospitals. Out of 124 health personnel in the entire district, 77 (62.1 %) were interviewed. Fifty two (67.5 %) were females and 25 (32.5 %) males. Their categories were; 24 (33.8 %) state registered nurses, 18 (25.4 %) nursing aids, 4 (5.6 %) laboratory technicians, 2 (2.8 %) midwives and 23 (32.4 %) were personnel of other categories such as community agents and community pharmacy attendants.

### Frequency and planning of vaccinations sessions

All the health facilities organized vaccination at fixed post (in the health facility). Regarding the frequency of organizing vaccination, 4 (9.5 %) health facilities program at least four sessions monthly, equivalent to an average of one vaccination session per week as seen on Table 1. A total of 2 (4.8 %) health facilities programed an outreach session within the last 3 months. A total of 27 (64.3 %) health facilities had a micro plan for vaccination activities. From Table 1, 24 (32.9 %) health personnel had not been supervised for the last 6 months prior to the study.

**Table 1:** Frequency, Planning and supervision of vaccination activities

Determinants	Availability		
	Yes. (%)	No. (%)	*p*-value
Health facilities that Organize vaccination activities	42 (100)	0	-
Health facilities that have a vaccination micro plan	27 (64.3)	15 (35.7)	0.07
Health facilities that programmed outreach vaccinations within last 3 months.	2 (4.8)	40 (95.2)	0.00
Frequency of Programming Vaccination Sessions in Health facilities	Number of Health facilities		Percentage (%)
Monthly	29		69.0
Two times a month	5		11.9
Three times a month	4		9.5
At least four times a month (equivalent to 1 per week)	4		9.5
Total	42		100
Frequency of supervision received by health personnel within last 6 months	Number of Health personnel		Proportion (%)
Did not receive any supervision	24		32.9
Received one supervision	33		45.2
Received at least two supervisions	16		21.9
Total	73		100

*P*-value < 0.05 indicates strong evidence of a difference

### Availability of essential resources and tools for vaccination activities

From Table 2, 7 (16.7 %) health facilities did not have a functional refrigerator, 23 (54.8 %) did not have a transportation machine, 28 (80 %) did not have a contingency plan and 9 (21.4 %) did not have electricity. Regarding alternative sources of power supply, 5 (11.6 %) health facilities had alternative sources of power supply which were; solar energy 1 (2.3 %), and a generator 4 (9.3 %). A total of 5 (11.9 %) health facilities had run short of stock of vaccines within the last 3 months. The primary causes of the shortages were due to; delay in provision of stock 8 (88.9 %) and rupture of the cold chain 1 (11.1 %). Regarding the frequency for ordering vaccines, 24 (31.2 %) said they order vaccines weekly, 34 (44.2 %) said they order vaccines monthly while 19 (24.7 %) order vaccines after several months.

**Table 2:** Availability of Resources in Health facilities

Availability of resources in health facilities	Availability		*P*-value
	Yes (%)	No (%)	
Functional refrigerator (*n* = 42)	35 (83.3)	7 (16.7)	0.00
Existence of temperature chart on Fridge (*n* = 35)	24 (68.6)	11 (31.4)	0.04
Presence of thermometer in Fridge(*n* = 35)	29 (82.9)	6 (17.1)	0.00
Have a contingency plan (*n* = 35)	7 (20)	28 (80)	0.00
Availability of ice parks (*n* = 35)	29 (82.9)	6 (17.1)	0.00
Availability of vaccine order form (*n* = 35)	32 (91.4)	3 (8.6)	0.00
Run out of stock for vaccines (*n* = 42)	5 (11.9)	37 (88.1)	0.00
Availability of a vaccine management register (*n* = 35)	27 (77.1)	8 (22.9)	0.01
Have electricity as main source of power (*n* = 42)	33 (78.6)	9 (21.4)	0.00
Have transportation machine (vehicle or motorcycle) (*n* = 42)	19 (45.2)	23 (54.8)	0.55
Availability of vaccine carrier (*n* = 42)	41 (97.6)	1 (2.4)	0.00
Availability of the EPI SOP guideline (*n* = 42)	30 (71.4)	12 (28.6)	0.01

*P*-value < 0.05 indicates strong evidence of a difference

### Knowledge of health personnel on vaccines and cold chain management

Regarding knowledge about antigens that are eligible to the four weeks open vial policy, 32 (43.8 %) identified all three antigens which includes; pentavalent, anti-tetanus and OPV as seen on Table 3. Others included in the list, BCG 12 (16.4 %), VAA/VAR (yellow fever and Measles) 17 (23.3 %) and other antigens 14 (19, 2 %). Regarding the management of the cold chain, 50 (68.5 %) knew the appropriate temperature limits required to conserve vaccines (2 to 8 °C) at the health facility level. Regarding the knowledge about vaccines that can be frozen without being damaged, 3 (4.1 %) identified all the three antigens which includes OPV, measles and BCG as seen on Table 3. Regarding their knowledge on measures to be take in order to preserve the potency of vaccines in case of a power failure that last for more than 2 days; 27 (36.5 %) said they will empty the fridge, 3 (4.1) said they will throw the vaccines away, 3 (4.1 %) said they will wait until power supply is restored, 37 (50 %) said they will carry the vaccines to a neighboring health facility that have power supply, and 11 (14.9 %) said they will call the district health service for help.

**Table 3:** Health personnel Knowledge on vaccine and cold chain

Determinants	Yes (%)	No (%)	*P*-value
Know the appropriate temperature limits required to conserve vaccines (2 to 8 °C) in the refrigerator at the health facility	50 (68.5)	23 (31.5)	0.00
Knowledge of health personnel on antigens that are eligible for the four weeks open vile policy	Yes (%)	No (%)	
Pentavalent (DPT-HepB + Hib) vaccine	52 (71.2)	21 (28.8)	0.00
Anti-Tetanus vaccine	61 (83.6)	12 (16.4)	0.00
Oral Polio Vaccine (OPV)	42 (57.5)	31 (42.5)	0.21
All three antigens above (pentavalent, anti-tetanus and OPV)	32 (43.8)	41 (56.2)	0.29
BCG vaccine	12 (16.4)	61 (83.6)	0.00
Measles/Yellow Fever (VAA/VAR)	17 (23.3)	56 (76.7)	0.00
Other antigens	14 (19.2)	59 (80.8)	0.00
Knowledge of health personnel on vaccines that can be frozen without being damaged.	Yes (%)	No (%)	
Oral Polio Vaccine (OPV)	19 (26.0)	54 (74.0)	0.00
Measle Vaccine	17 (23.3)	56 (76.7)	0.00
BCG	12 (16.4)	61 (83.6)	0.00
All three antigens listed above (OPV, measles and BCG)	3 (4.1)	70 (95.9)	0.00
Knowledge of personnel on Diseases under epidemiological surveillance targeted by the Expanded Program on Immunization	Yes (%)	No (%)	
Yellow Fever	59 (80.8)	14 (19.2)	0.00
Acute Flaccid Paralysis	4 (5.5)	69 (94.5)	0.00
Measles	66 (90.4)	7 (9.6)	0.00
Neonatal Tetanus	57 (78.1)	16 (21.3)	0.00
All four mentioned above	3 (4.1)	70 (95.9)	0.00

*P*-value < 0.05 indicates strong evidence of a difference

### Knowledge of health personnel on diseases under epidemiological surveillance targeted by the expanded program on immunization

Regarding their knowledge on diseases under epidemiological surveillance in the EPI, 3 (4.1 %) personnel identified all the four diseases which includes yellow fever, acute flaccid paralysis, measles and neonatal tetanus as seen on Table 3. For example, regarding their knowledge on the identification of a suspected case of measles, 65 (84.4 %) said fever, 71 (92.2 %) said rashes and 60 (77.9 %) said conjunctivitis. In total, 50 (64.9 %) identified all the three clinical signs and symptoms. Concerning the type of biological sample to be collected from suspected patient for laboratory investigation, 52 (67.5 %) said serum.

## Discussions

This was a descriptive situational study to assess the provision of immunization service with regards to the frequency and strategy of vaccination adopted, the knowledge of health personnel (on cold chain management and on diseases targeted by the EPI) and the availability of essential resources and tools necessary for an adequate provision of vaccination services in the Dschang Health district (West Cameroon).

Our results show that, most health facilities 29 (69.0 %) organize one vaccination session monthly, only 2 (4.8 %) health facilities had organized an outreach strategy within the last 3 months prior to our study, 15 (35.7 %) health facilities did not have a vaccination micro plan, 24 (32.9 %) health personnel had not been supervised for at least the last 6 months, 7 (16.7 %) health facilities did not have a functional refrigerator,1 (2.4 %) did not have a vaccine carrier, 23 (54.8 %) did not have a transportation machine such as a vehicle or motorcycle and 12 (28.6 %) did not have an EPI guideline. The knowledge of health personnel on vaccine and cold chain management and on diseases under epidemiological surveillance by the EPI was found to be very limited.

Provision of vaccination services at fixed posts and outreach posts is a backbone of a successful and sustainable vaccination system especially in developing countries [16]. According to the EPI guideline in Cameroon, health facilities that have a functional refrigerator are expected to organize vaccination sessions daily whereas those without a fridge vaccinate twice a week. It also recommends the use of outreach and mobile vaccination strategies in order to reach target populations who live farther than 5 and 10Km respectively from a health facility that offer vaccination services [7]. From these results, it is clear that health personnel did not adhere to recommendations of the guideline as far as the frequency and strategy of vaccination is concerned.

Frequent provision of vaccination requires constant availability of vaccines, availability of qualified health personnel, sensitization of parents to bring their children for vaccination, motivation of the health personnel and the constant availability of materials and tools [11–14]. Addressing all these elements simultaneously to ensure frequent provision of vaccination has been a difficult task for most districts heads and chiefs of centers in health facilities. As a result of this, most health facilities will program one vaccination session a month, while only a few manage to program at least two sessions monthly even when population demand for vaccination is high. We also noted the lack of organization of outreach strategies, given that about 25 % of the EPI target populations in this district live further than 5Km from a health facility. This implies that for a parent to get his/her child vaccinated, they will have to travel long distances in order to reach health facilities to get their child vaccinated. Though it is worth the sacrifice for these parents, doing this regularly is costly, tiring and inconvenient. Consequently, they may not always be able to meet up with vaccination schedules. On the other hand, an outreach strategy is expensive; it requires transportation machines and motivated personnel to carry out this task. In addition, the topography of the Dschang health district is comprised of hills and slopes, with muddy and slippery roads especially in rainy seasons which makes outreach vaccination even more challenging. In some villages, the only possible means of transportation is a motorcycle or bicycle. Given that most health centers in the district have only one or two health personnel in the facility, conducting outreach strategies may have serious impacts on the other activities offered in the health facility, since they are also the ones involved in providing other health care services in the facility. However, there appear to be good preparedness for outreach strategies with regards to the availability of tools and resources..We noted that 95.3 % of health facilities had a vaccine carrier with ice packs (82.9 %). This is an indication that the lack of health personnel, lack of funding, the unavailability of transport logistics and the lack of motivation of personnel are the suspected primary reasons hindering the organization of outreach vaccination sessions to cover the hard to reach populations. Similar results were obtained in a study conducted in Mozambique [17], indicating that lack of funding and logistics were the primary factors responsible for non-implementation of outreach vaccinations.

Vaccines are sensitive biological substances that gradually lose their potency with time and this loss of potency can be accelerated when stored out of the recommended temperature range [18, 19]. To achieve a successful immunization, there is a need for an effective cold chain system for the storage and transportation of vaccines in a way that their potency is preserved. Findings of this study have indicated that there are still health facilities that do not have a functional refrigerator, while some facilities that have a refrigerator are lacking accessories for appropriate cold chain and vaccine management; such as thermometers, a temperature chart and alternative power supply. This is an indication of limited resource availability for vaccine and cold chain management. Similar results were obtained by other studies conducted in other parts of the country [11, 13]. That may also explain why some health facilities ran out of vaccine stock due to lack of storage capacity.

The knowledge of health personnel on vaccines management, cold chain management and on diseases under epidemiological surveillance by the EPI was found to be very limited. Following recommendations from WHO [20], the four weeks open vial policy is applicable in Cameroon, which consists of preserving open vials of DPT-HepB + Hib, antitetanus vaccine and OPV for four weeks until they are used with the conditions that (they have not expired, not contaminated, was not exposed to extreme heat or cold, was not immersed in water, and if the Vaccine Vial Monitor (VVM) is good. On the other hand, antigens such as BCG, VAR, and VAA cannot be conserved for more than 6 hours once they have been opened and must be discarded. Knowledge on the application of this policy will not only reduce wastage rates but will also help the personnel to better plan a vaccination session and to ensure quality immunization service delivery. It is clear from our study that personnel do not master the application of the open vial policy with respect to the different antigens. This may results to wrong application of the policy to the wrong antigen, leading to loss of vaccine potency, increase wastage rates, post immunization adverse events and failure of the vaccination program. We equally noted that most personnel do not master how to arrange vaccines in the refrigerator based on their sensitivity to temperature. Given that the sensitivity to temperature of the different antigens varies, care needs to be taken when placing vaccines in the refrigerator. Less sensitive vaccines to cold (BCG, OPV, VAR) are placed closest to the freezing compartment while more sensitive ones are placed beneath. The temperature of the fridge also need to be controlled between 2 and 8 °C. From the study, the knowledge of health personnel on arranging vaccines in the refrigerator was found to be limited. Similar results were obtained by other studies conducted in other parts of the country [11, 13].

Regarding the knowledge of health personnel on the four diseases under surveillance in the EPI, we found out that their knowledge was limited. Personnel showed little mastery regarding the identification of the diseases through their case definition and in the surveillance procedure of these diseases. The surveillance of these diseases has been integrated in the MPA offered in health centers and health personnel are expected to master these diseases and the procedure of their surveillance. Very few personnel could identify all the four diseases which are Yellow fever, Acute Flaccid Paralysis, Neonatal Tetanus and Measles. The least known among the four diseases was Acute Flaccid Paralysis while the knowledge on the other three diseases; measles, yellow fever and neonatal tetanus was acceptable. The limited knowledge on these diseases can eventually result to missing of cases during surveillance. Consequently, suspected cases will not be quickly identified, and reported. This will result to spreading of the disease and outbreaks of epidemics as the case of the wild polio virus epidemic in 2013.

Most of the above mentioned weaknesses can readily be corrected and improved. One key strategy to improve on them is regular supervision and the ownership and use of the EPI guideline. The EPI guideline recommends regular supervision to ensure that what is planned is actually implemented [7]. And this supervision is mainly to correct inconsistencies. Supervision can improve the knowledge of health personnel on diseases under surveillance, and on vaccine and cold chain managements [21, 22]. Unfortunately, the frequency of supervision in the district is very low. Many health facilities had not been supervised for the last 6 months. This is because a successful supervision is expensive as it requires logistics (cars or motorcycle with fueling), motivation of the supervisors and expertise which most of the time suffer limited funding. Consequently, supervisors will prefer to supervise only health facilities that are close while those in hard to reach areas will remain unsupervised. Similarly, the EPI national guideline contains instructions and procedures among others on the management of vaccines, cold chain and on the surveillance of diseases. Most health facilities did not have this guideline. Though the ownership of the guideline does not guarantee its use, we believe that it can significantly improve EPI service delivery, especially when it is incorporated with regular supervision.

The main limitation of this study is that at the time of interview, in some facilities we did not meet the main personnel in charge of vaccination activities in the facility. However, we replaced him by selecting other personnel from the same health facility, who also participate in vaccination activities. Besides this, we believe that the study is sound.

## Conclusion

The frequency and strategic provision of immunization services in the Dschang Health district is inadequate. There is low frequency of organization of vaccinations both at fixed posts at the health facilities and for outreach or mobile vaccinations strategies. There is lack of resource availability such as lack of personnel, refrigerators, transportation machines, and funding, required for an adequate provision of immunization services. The knowledge of health personnel on vaccine management such as application of the open vile policy, on cold chain management such as correct arrangement of vaccines in the refrigerator based on their sensitivity to temperature and their knowledge on diseases under surveillance by the EPI is limited. The frequency of supervision is very low. Corrective measures should include among others; increase frequency of supervision of EPI activities in order to constantly correct inconsistencies in immunization services delivery practices, to ensure personnel adhere to recommendations of the EPI guideline, to update the knowledge of health personnel on the surveillance of diseases targeted by the EPI, and their knowledge on vaccine and cold chain management. All health facilities should be provided with refrigerators, EPI guidelines should be provided to personnel, logistics materials such as motorcycles should be provided to facilitate transportation during outreach vaccinations, health facilities should be provided with extra funding to used to motivation health personnel and to afford fuel to permit them conduct outreach vaccinations in hard to reach areas. We also recommend similar studies to be conducted in other districts and other studies to determine factors and determinants resulting to poor immunization service delivery. This will help to improve the quality of immunization service delivery in the district and in the entire country, and eventually reduce outbreaks of future epidemics.

## Abbreviations

BCG, tuberculosis vaccine; DHS, district health service; DPT-HepB + Hib, diphtheria, pertusis tetanus, hepatitis B vaccine; EPI, expanded program on immunization; IHC, integrated health center; MPA, minimum package of activities; OPV, oral polio vaccine; PHC, primary health care; VAA, yellow fever vaccine; VAR, measles vaccine; WHO, World Health Organization

## Additional files

Additional file 1: Dataset. (XLSX 86.4 kb) Additional file 2: Health facility questionnaire. (PDF 380 kb) Additional file 3: Health personnel. (PDF 298 kb)

## References

[1] Mackroth MS, Irwin K, Vandelaer J, Hombach J, Eckert LO (2010). Immunizing school-age children and adolescents: experience from low- and middle-income countries. Vaccine.

[2] Immunization Basics, US Agency for International Development (USAID). WHO: periodic intensification of routine immunization. 2009. http://www.immunizationbasics.jsi.com/Docs/PIRImonograph_Feb09.pdf.

[3] World Health Organization: Global programme for vaccines and immunization: expanded programme on immunization. Safe vaccine handling, cold chain and immunizations. Geneva: 1998. http://www.old.health.gov.il/download/forms/a3039_GDPv.pdf.

[4] United Nations inter-agency Group for Child Mortality Estimation (IGME) (2013). Levels and Trends in Child Mortality Report, 2013.

[5] Ministry of Public Health. Decision n° 0333/MSP/CAB of July 29, 2002 reorganizing the expanded program of immunization in Cameroon. [http://acdevcm.free.fr/sante/pev.html]

[6] Ministry of Health. The 2011–2015 Comprehensive Multiyear plan For the Expanded Immunization Programme Cameroon. Cameroon: Ministry of Public Health; May 2011.

[7] Ministry of Health. The Expanded Program on Immunization Norms and Standards Guideline. Cameroon: Ministry of Public Health; 2009.

[8] Ministry of Health. Cameroon National Demographic and Health Survey (NDHS). National Institute of Statistics. Cameroon: Ministry of Public Health Cameroon report. 2012.

[9] World Health Organization, WHO. Global Alert and Response (GAR), Disease outbreak news: Wild poliovirus in Cameroon. 21 November 2013. [http://apps.who.int/csr/don/2013_11_21/en/index.html.

[10] Ebong CE, Levy P (2011). Impact of the introduction of new vaccines and vaccine wastage rate on the cost-effectiveness of routine EPI: lessons from a descriptive study in a Cameroonian health district. Cost Eff Resour Alloc.

[11] Ateudjieu J, Kenfack B, Nkontchou BW, Demanou M (2013). Program on immunization and cold chain monitoring: the status in eight health districts in Cameroon. BMC Res Notes.

[12] Wallace A (2012). Strengthening Evidence-Based Planning of Integrated Health Service Delivery Through Local Measures of Health Intervention Delivery Times. J Infect Dis.

[13] Yakum MN, Ateudjieu J, Walter EA, Watcho P (2015). Vaccine storage and cold chain monitoring in the North West region of Cameroon: a cross sectional study. BMC Res Notes.

[14] Gianluca Russo, Alessandro Miglietta, Patrizio Pezzotti, Rodrigue Mabvouna Biguioh, Georges Bouting Mayaka, Martin Sanou Sobze, Paola Stefanelli, Vincenzo Vullo and Giovanni Rezza. Vaccine coverage and determinants of incomplete vaccination in children aged 12–23 months in Dschang, West Region, Cameroon: a cross-sectional survey during a polio outbreak. doi.10.1186/s12889-015-2000-2 10.1186/s12889-015-2000-2 PMC4496879 26156158

[15] Owona Essomba R, Bryant M, Bodart C (1993). The reorientation of primary health care in Cameroon: Rationale, obstacles and constraints. Health Policy Plan.

[16] Streefland PH, Chowdhurry AMR, Ramos P (1999). Quality of Vaccination Services and Social Demand For Vaccination in Africa and Asia. Bull World Health Organ.

[17] WHO. Country cooperation strategy; Mozambique; 2009–2013.

[18] World Health Organization (1998). Global programme for vaccines and immunization: expanded programme on immunization: safe vaccine handling: cold chain and immunizations.

[19] World Health Organization (2004). Immunization in practice: module 3: the cold chain.

[20] WHO. Gérer les équipements de la chaîne du froid. Guide à l’intention des responsables nationaux de la logistique. WHO/EPI/LHIS/96.02.

[21] Som M, Panda B, Pati S, Nallala S, Anasuya A, Chauhan A, Sen S, Ashish K, Zodpey S (2014). Effect of Supportive Supervision on Routine Immunization Service Delivery-A Randomized Post-Test Study in Odisha. Glob J Health Sci.

[22] Djibuti M, Gotsadze G, Zoidze A, Mataradze G, Esmail L, Kohler C, Jillian C (2009). The role of supportive supervision on immunization program outcome - a randomized field trial from Georgia. BMC Int Health Hum Rights.

