# Supplementary material for: The expanded program on immunization service delivery in the Dschang health district, west region of Cameroon: a cross sectional survey
Source: BMC Public Health. 2016 Aug 17;16:801. doi: 10.1186/s12889-016-3429-7 (PMC4987984; doi:10.1186/s12889-016-3429-7)
Supplement: Additional file 2: — Health facility questionnaire. (PDF 380 kb) [file 12889_2016_3429_MOESM2_ESM.pdf]

REPUBLIQUE DU CAMEROUN

PAIX -TRAVAIL- PATRIE

****

UNIVERSITE DE DSCHANG

****

DEPARTEMENT DE SCIENCES BIOMEDICALES

REPUBLIC OF CAMEROON

PEACE- WORK-FATHERLAND

*****

UNIVERSITY OF DSCHANG

*****

DEPARTMENT OF BIOMEDICAL SCIENCE

Questionnaire For Health Facility

Code:I__I__I__I__I

Immunization Service Delivery in the Dschang Health District

Hello,

You are invited to participate in this study conducted by students in the department of Biomedical Sciences of the University of Dschang, Cameroon. The aim of this study is to assess immunization service delivery in the Dschang Health district. These includes, assessing the availability of essential tools and resources necessary for an adequate immunization service delivery, the vaccination strategies adopted and the knowledge of health personnel on vaccine and cold chain management. Once you accept to participate, you will be interviewed by a member of the study team for about 15 minutes. Information you provide will be collected on questionnaires. The study team member will also make some direct visual observations in your health facility to identify the availability or non-availability of some materials or tools. All information that you provide the research team member, shall be recorded anonymous, kept strictly confidential and shall not be shared with a third party. You shall not be paid for participating in this study. You may feel free to refuse participating or to continue participating in the study at any time. If you have any questions about the study or have any problems participating, please you may contact Dr Jérôme Ateudjieu at the University of Dschang through the number 699701011, or email, jateudj@yahoo.fr.

Signature of Participants :…………………………………………..Date : _ _ / _ _ / _ _ _ _

Signature of surveyor : …………………………………………… Date : _ _ / _ _ / _ _ _ _

I- IDENTIFICATION

Consent to participate Yes ☐ No ☐

Heath Area :……………………………………………………………………………………

Name of Health Facility ____

Type of Health Facility: Public □ Private □ Confessional□

Category of Health Facility: District Hospital (DH) □, sub-divisional hospital (CMA) □ Integrated health Centre (IHC) □ other □ (precise) ______________

Table 1 : Target Population per health Area

Village	Total Pop (p1)	0-11 months (4% x P1)	Pregnant women (5% x P1)	Distance from closest health facility (km)	Vaccination strategy adopted[1]	Number of Sessions planned/sessions executed
[1] A : fixed vaccination posts ; B : Outreach vaccination ; C: Mobile strategy						

Number of supervisions received for the last six months ____________________

II. Availability of Tools and Resources.

Table 2 : Resources availability

Equipements	Number Available	Number Functional	Brand/Mark
Refrigerator			
Accumulators			
accumulateur			
Thermometer in Refrigerator			
Temperature Chart			
Case definition forms			
Vehicle			
Motorcycle			
Bicycle			
Generator			
Solar Panel			

Table 3 :Observation Parameters

**Indicators**		
Presence of Thermometer in the refrigerator?	Yes	No
Reading of thermometer at the time of verification(^oC)		
Number of temperature recorded higher than 8 ^oC for the last 3 months		
Number of temperature recorded lower than 2 ^oC for the last 3 months		
Is the temperature Chart completely filled?	Yes	No
Contigency plan available	Yes	No
Presence of other substances in the refrigerator ?	Yes	No
Availability of EPI guideline in the health facility ?	Yes	No
Power failures in the last month?	Yes	No

1. Main source of Energy in health facility (a) électricité (b) kérosène (c) gaz (d) générateur (e) rayons soleil
2. Maximum duration of power failure in the last 3 months________

III. Other Parameters Verified

Parameters	Yes	No
Vaccination microplan present ?		
Case definition forms for measles available ?		
Case report form for polio present ?		
Sample collection guideline forms available ?		
Vaccine Management Register available		
Vaccine command form present ?		
