# Supplementary material for: The expanded program on immunization service delivery in the Dschang health district, west region of Cameroon: a cross sectional survey
Source: BMC Public Health. 2016 Aug 17;16:801. doi: 10.1186/s12889-016-3429-7 (PMC4987984; doi:10.1186/s12889-016-3429-7)
Supplement: Additional file 3: — Health personnel. (PDF 298 kb) [file 12889_2016_3429_MOESM3_ESM.pdf]

REPUBLIQUE DU CAMEROUN

PAIX -TRAVAIL- PATRIE

****

UNIVERSITE DE DSCHANG

****

DEPARTEMENT DE SCIENCES BIOMEDICALES

REPUBLIC OF CAMEROON

PEACE- WORK-FATHERLAND

*****

UNIVERSITY OF DSCHANG

*****

DEPARTMENT OF BIOMEDICAL SCIENCE

Questionnaire Health Personnel

Code:I__I__I__I__I

Immunization Service Delivery in the Dschang Health District

Hello,

You are invited to participate in this study conducted by students in the department of Biomedical Sciences of the University of Dschang, Cameroon. The aim of this study is to assess immunization service delivery in the Dschang Health district. These includes, assessing the availability of essential tools and resources necessary for an adequate immunization service delivery, the vaccination strategies adopted and the knowledge of health personnel on vaccine and cold chain management. Once you accept to participate, you will be interviewed by a member of the study team for about 15 minutes. Information you provide will be collected on questionnaires. The study team member will also make some direct visual observations in your health facility to identify the availability or non-availability of some materials or tools. All information that you provide the research team member, shall be recorded anonymous, kept strictly confidential and shall not be shared with a third party. You shall not be paid for participating in this study. You may feel free to refuse participating or to continue participating in the study at any time. If you have any questions about the study or have any problems participating, please you may contact Dr Jérôme Ateudjieu at the University of Dschang through the number 699701011, or email, jateudj@yahoo.fr.

Signature of Participants :…………………………………………..Date : _ _ / _ _ / _ _ _ _

Signature of surveyor : ……………………………………………. Date : _ _ / _ _ / _ _ _ _

I- IDENTIFICATION

Consent to participate? Yes ☐ No ☐

Health Area:………………………………………………………………………………

Name of health facility : ____

Type of health facility : Public□ Private□ Confessionnal□

Category of health facility: District hospital(DH) □,Sub-divisional hospital(CMA) □ Integrated health centre (IHC) □ Others □ (specify) ______________

Sex : Male □ Female□

Qualification of personnel: Medical Doctor □ State registered nurse □ Mid wife □ Nursing aid □ lab technician □ Others □ (specify) _______________

Health Service _______________

II. Vaccine Management

1- How often do you make command for vaccines : Weekly □ Monthly □ every Semester□
2- Have you had rupture in stock of vaccines within the last three months? Yes □ No □
3- If yes, how many times? ____
4- What antigen had the stock rupture? __________________
5- What was the duration of the rupture? _________________________
6- What caused the rupture? Delay in supply □ ; Due to high demand □ ; Problem of the cold chain □ Others □ (specify) ______________________________
7- Which of the following opened viles of vaccines can be used again after a vaccination session?

VAT□ ; DTC/HepB□ ; VPO□ ; BCG□ ; VAA/VAR□ ; None□ ; I don't know □

II. Cold Chain Management and Logistics

1-What are the measures to take in case of a power failure lasting longer than 48 hours?

Empty the fridge □ ; Complain to the district □ ; Discard the vaccines □ ; Carry vaccines to nearest health facility with power □ ; Keep waiting □

2-What is the recommended temperature to save vaccines?

above +8°C□ ; Between +2°C et +8°C□ ; Below +2°C□

3-Identify the vaccines or antigens that can be frozen without being damaged.

BCG□ ; VAT□ ; VAA□ ; VPO□ ; VAR □ DTC/Hép B□

III. Surveillance of Diseases targted by the Expanded program on Immunization

1- Identify the diseases targeted by the EPI program?

Tuberculoses □ ; Neonatal tetanus □ ; Poliomyelitis □ ; Measles □ Hepatitis B □ ; Yellow Fever □ ; Cholera □ ; Typhoid fever □ ; HIV/AIDS □

2- Identify among the following elements the signs for a suspected case of measles in children under 5 years?Fever □ ; Acute Flaccid Paralysis □ ; Diarrhea □ Rashes □ ; Conjunctivities □
3- Identify the specimens to collect from a suspected measles patients for laboratory findings?

Cerebro Spinal fluid □ ; Saliva □ ; Serum □; Urine □ ; Other (specify) ________________________________

IV. Programing and Supervision of Vaccination Activities

1- Do you have a vaccination micro plan? Yes □ No □
2- If Yes, Visual verification and confirmation? Yes □ No □
3- Number of vaccination sessions organized per week? _________
4- Number of Vaccination Sessions organized per month? _________
5- Number of outreach vaccination sessions organized in last 6 months? ______
6- Number of supervisions received in the last six months? _________
